# Supplementary figures and images for: Long-term alfalfa planting mediates the coupling of soil water and organic carbon storage in a semi-arid area of the Loess Plateau, China
Source: PeerJ. 2024 Nov 5;12:e18373. doi: 10.7717/peerj.18373 (PMC11546141; doi:10.7717/peerj.18373)

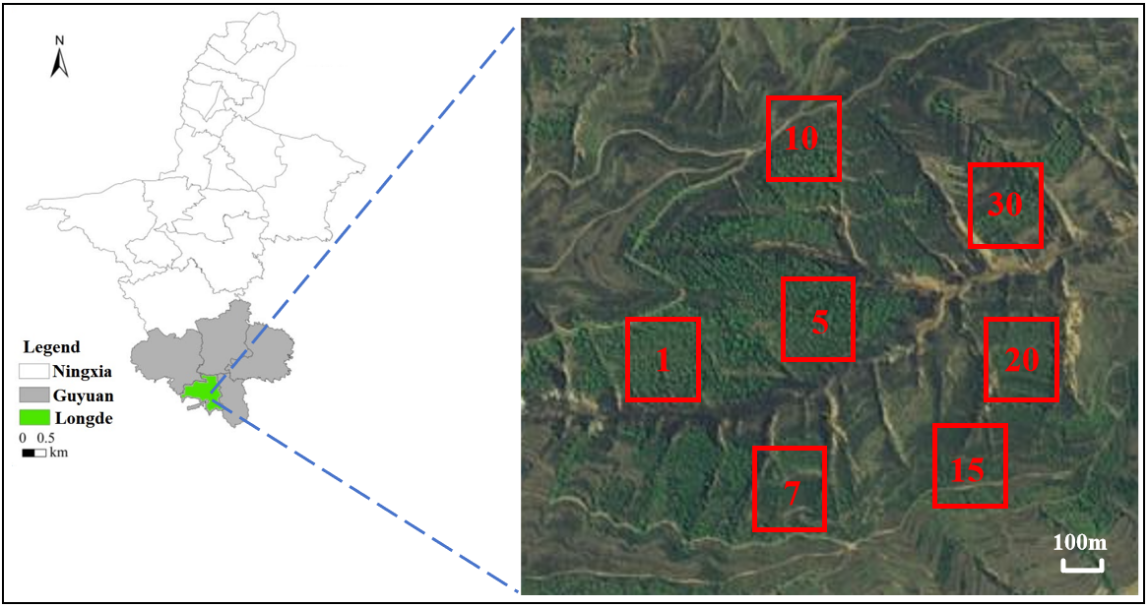

Supplement: Figure S1 — Image source: http://bzdt.ch.mnr.gov.cn/index.html. [file peerj-12-18373-s001.png]

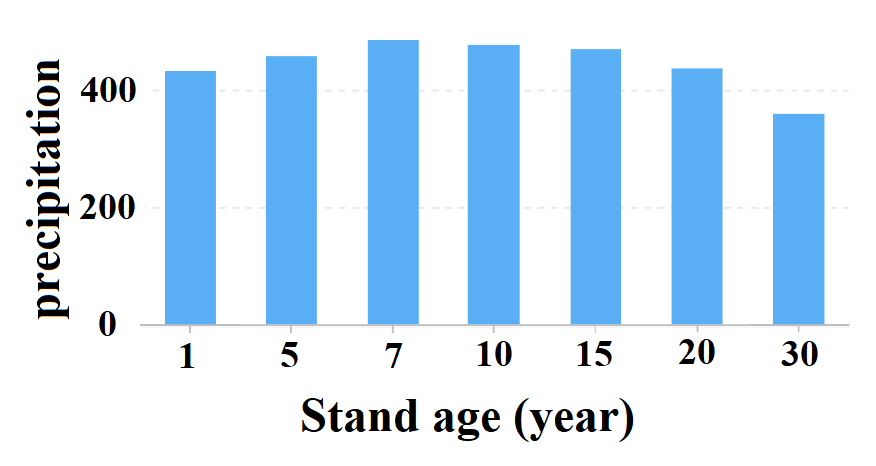

Supplement: Figure S2 [file peerj-12-18373-s002.png]

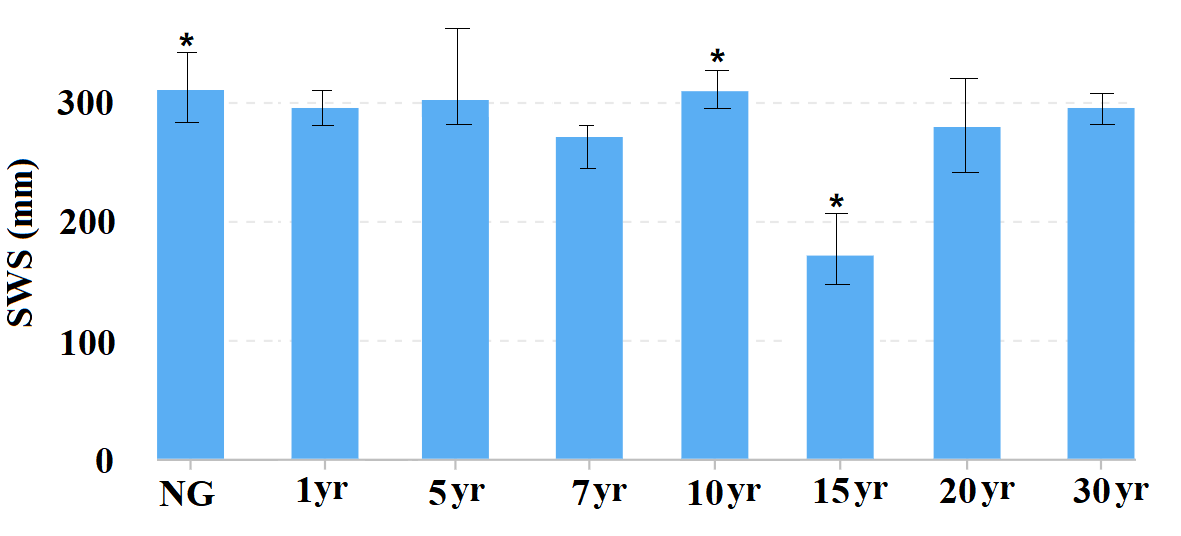

Supplement: Figure S3 — NG means natural grassland. * indicate significant difference in group means among treatment at the 0.05 level. [file peerj-12-18373-s003.png]
