# Supplementary material for: Long-term alfalfa planting mediates the coupling of soil water and organic carbon storage in a semi-arid area of the Loess Plateau, China
Source: PeerJ. 2024 Nov 5;12:e18373. doi: 10.7717/peerj.18373 (PMC11546141; doi:10.7717/peerj.18373)
Supplement: Table S1 [file peerj-12-18373-s004.docx]

| Stand age (year) | Dominant species | Plant density (plants/m^2^) | Importance value | Associated species |
| --- | --- | --- | --- | --- |
| 1 | *Medicago sativa* | 97 | 0.53 | *Chenopodium aristatum* |
|  | *Setaria viridis* | 27 | 0.13 | *Geranium wilfordii* |
|  | *Chenopodium glaucum* | 14 | 0.15 | *Convolvulus arvensis* |
| 5 | *Medicago sativa* | 100 | 0.36 | *Plantago asiatica* |
|  | *Tripolium vulgare* | 88 | 0.23 | *Viola philippica* |
|  | *Artemisia scoparia* | 86 | 0.15 | *Leymus secalinus* |
| 7 | *Medicago sativa* | 113 | 0.42 | *Poa annua* |
|  | *Artemisia scoparia* | 70 | 0.30 | *Plantago asiatica* |
|  | *Agropyron cristatum* | 89 | 0.15 | *Saussurea japonica* |
| 10 | *Medicago sativa* | 96 | 0.40 | *Viola philippica* |
|  | *Artemisia scoparia* | 60 | 0.27 | *Cirsium japonicum* |
|  | *Saussurea japonica* | 5 | 0.15 | *Plantago asiatica* |
| 15 | *Medicago sativa* | 90 | 0.23 | *Plantago asiatica* |
|  | *Poa annua* | 11 | 0.16 | *Ixeris chinensis* |
|  | *Stipa bungeana* | 50 | 0.10 | *Artemisia scoparia* |
| 20 | *Medicago sativa* | 30 | 0.22 | *Leymus secalinus* |
|  | *Poa annua* | 20 | 0.24 | *Tripolium vulgare* |
|  | *Stipa bungeana* | 7 | 0.14 | *Allium mongolicum* |
| 30 | *Medicago sativa* | 9 | 0.14 | *Saussurea japonica* |
|  | *Viola philippica* | 7 | 0.12 | *Convolvulus arvensis* |
|  | *Stipa bungeana* | 26 | 0.60 | *Stipa breviflora* |
